# A Bayesian Entropy Approach to Sectoral Systemic Risk Modeling

**DOI:** 10.3390/e22121371

**Published:** 2020-12-04

**Authors:** Radu Lupu, Adrian Cantemir Călin, Cristina Georgiana Zeldea, Iulia Lupu

**Affiliations:** 1 Department of International Business and Economics, Bucharest University of Economic Studies, 010404 Bucharest, Romania; radu.lupu@rei.ase.ro (R.L.); cristinazeldea@iem.ro (C.G.Z.); 2 Institute for Economic Forecasting, Romanian Academy, 050711 Bucharest, Romania; 3 Institute for World Economy, Romanian Academy, 050711 Bucharest, Romania; 4 “Victor Slăvescu” Centre for Financial and Monetary Research, Romanian Academy, 050711 Bucharest, Romania; iulia.s.lupu@gmail.com

**Keywords:** systemic risk measurement, spillovers, entropy measures, Bayesian inference, economic sectors

## Abstract

We investigate the dynamics of systemic risk of European companies using an approach that merges paradigmatic risk measures such as Marginal Expected Shortfall, CoVaR, and Delta CoVaR, with a Bayesian entropy estimation method. Our purpose is to bring to light potential spillover effects of the entropy indicator for the systemic risk measures computed on the 24 sectors that compose the STOXX 600 index. Our results show that several sectors have a high proclivity for generating spillovers. In general, the largest influences are delivered by Capital Goods, Banks, Diversified Financials, Insurance, and Real Estate. We also bring detailed evidence on the sectors that are the most pregnable to spillovers and on those that represent the main contributors of spillovers.

## 1. Introduction

Despite the lack of general agreement on a plain, ubiquitous definition, systemic risk lures the attention of regulators and consequently all market participants. A wide range of indicators accounts for the various facets of systemic risk and they generally capture loss behavior and connections.

The concept of systemic risk has received a heavy dose of both theoretical and empirical attention, given its character and the realities observed during the most recent financial crisis. An area of consensus in the literature germinates on the idea that the root of systemic risk derives from the intricate web of interconnections endemic to modern financial institutions that acts as a propagation channel for a given shock—see for example Wang et al. [1] or Gofman [2]. The general factors considered to be catalyzers of these linkages vary from investment networks and financial innovation to lending and trade agreements, see Gong et al. [3]. As pointed out by numerous studies among which we mention Yao [4], financial crises are times of grave systemic tensions in which financial stress is conveyed to the real economy translating into recession. Equally concerning are the associated phenomena which involve stock market crashes, monetary constriction, heavy oscillation in exchange rates, and pulsating volatility. Moreover, systemic risk is toxic for the real economy, because of its detrimental effects on GDP growth, production or the labor market [5].

If the above argumentation hints to the macroeconomic implications of systemic risk accumulation, it is worth mentioning that it has also microeconomic implications such as lowering diversification effects, see for example of Billio et al. [6].

As we will show below, the purpose of this manuscript is to put forward an entropy-based approach for systemic risk modeling and to provide an investigation on its spillover patterns. We are motivated by previous attempts at explaining various financial and risk management phenomena through the use of entropy such as Dimpfl and Peter [7], Huang, Shang, and Zhao [8], Niu and Wang [9], or more recently Pichler and Schlotter [10]. Despite this strand of literature, there is high sparsity in terms of empirical works that consider systemic risk via entropy measures. To the best of our knowledge, the only previous effort is the pioneering work of Billio et al. [6] that aims to construct an early warning indicator for systemic risk.

Our objective is to analyze the spillover effects of the entropy indicator for the systemic risk measures computed on the 24 sectors that compose the Stoxx 600 index. Billio et al. [6] provide evidence that cross-sectional entropy offers grounds for the setting of early warning systems in the banking sector. The present article contributes to the current literature in the following ways. Firstly, we employ all the sectors that compose the Stoxx 600, and not only financial institutions. Through this perspective, we aim to capture the source of shocks to the whole system and allow for other sectors (than the financial one) to generate these shocks. Secondly, for each day we compute the cross-entropy corresponding to each sector, for three systemic risk indicators. For instance, for the banking sector, we use 26 companies that compose this sector in the Stoxx 600 index. We therefore produce 24 time series of entropy indicators (corresponding to the 24 sectors) for each systemic risk measure, with a length of 3263 days. Thirdly, we employ the Diebold-Yilmaz [11] approach to account for spillover effects of these entropy indicators across the sectors. Another element that distinguishes our approach from previous related attempts is the use of a Bayesian entropy estimation as it will be shown in the dedicated section. The above-mentioned directions allow us to monitor and understand the patterns of systemic risk transmission across different sectors in the European financial markets.

The remainder of this paper is organized as follows. Section 2 offers a brief discussion on the related literature. Section 3 covers our computational approach. Section 4 exhibits the main results and implications, while Section 5 concludes.

## 2. Literature Review

Several fields of study have been using entropy intensively for a long time, but its use in finance has become of major interest rather recently. In recent years, the literature using entropy to grasp facets of uncertainty in financial systems has been growing. We can divide the research into three broad categories. The first category tackles the issue of informational complexity and efficiency of financial systems. Alvarez-Ramirez et al. [12] employ an approximate entropy method to find that market efficiency in the USA is time -variant and dependent on the frequency. They also prove that market efficiency is cyclical. Ortiz-Cruz et al. [13] analyze the informational efficiency and complexity of the oil market using a multiscale entropy approach. Their results show that the oil market has been generally efficient, with notable exceptions during global recessions, which implies that the risk of a crisis increases as informational efficiency decreases. Niu and Wang [9], and Casarin and Costola [14] test the effectiveness of entropy in conducting complexity analyses of high-frequency, or very large financial time series. The latter propose a Shannon entropy-based method of detecting structural changes in economic datasets. Risso [15] analyses differences in terms of informational efficiency between emerging and developed stock markets. Some Asian markets ranked first, while the least informationally efficient markets were those of ex-socialist countries.

The second strand of literature builds on the informational efficiency concept and focuses on information transmission, connectedness, or contagion per se, between financial systems. For example, Dimpfl and Peter [16] investigate information transfer between the CDS and corporate bond markets and find that information flows in both directions. However, the CDS market seems to dominate the bond market in what concerns credit risk, particularly during the crisis period. The same authors reiterate the transfer entropy method, on high-frequency data in order to identify different stock information transmission patterns between the US and EU before, during and after the global financial crisis [17]. The results also reveal a bidirectional flow. Interestingly, however, they show that the crisis represented a turning point: a decrease in the flows from the US, and an intensification of those from the EU. Junior et al. [18] also examine whether stock indices exert indirect influences on each other. They provide evidence that the aforementioned influences do exist, and the transfer entropy is an efficient method to quantify them. Maasoumi and Racine [19] also use entropy as a measure of dependence between stock indices and find significant dependence relations between the returns. Ji et al. [20] develop an entropy-based network to investigate information connectedness in housing markets. Several works rely on the framework of Diebold-Yilmaz [11] to test information transmission between pairs of countries—for example Ji et al. [20] Andrada-Félix et al. [21], Alter and Beyer [22], Chau and Deesomsak [23]. Our approach is somewhat similar in respect of using the Diebold-Yilmaz spillover index. Mistrulli [24] tests contagion in the Italian interbank market. He compares the entropy obtained results with the actual structure of interbank claims and finds that in some instances, the entropy method tends to overestimate contagion. Conversely, in a relatively similar study, Anand et al. [25] reach an opposite conclusion, such that the entropy tends to underestimate contagion in the interbank market.

The third category of studies, and perhaps the one that draws the nearest to our analysis, is addressing systemic risk explicitly and using various types of entropy frameworks to assess it. Ahn et al. [26] study the effects of stock market uncertainty -quantified through the Shannon entropy- on systemic risk and economic activities in China. Their study provides evidence that uncertainty shocks in stock markets determine an increase in systemic risk, and have a negative impact on economic fundamentals. Di Gangi et al. [27] investigate systemic risk in the banking system. Their cross-entropy-based method seems to be efficient in quantifying aggregated systemic risk and individual bank systemic importance. Several types of entropies have also proved to be useful predictive instruments for systemic risk. Gradojevic & Caric [28], for instance, show that the Tsallis and approximate entropies do have predictive power for systemic risk, but predictability depends to a great extent on the nature of the trigger event. Paltalidis et al. [29] address systemic risk in the euro area, stemming from the interbank, asset price and sovereign credit risk markets. Systemic risk is modeled in this case through the expected loss determined by contagion. Financial contagion is assessed by means of Maximum entropy, and results are that the euro area is highly vulnerable to contagion. Within the Eurozone, the banking systems of its periphery are more vulnerable and risk-prone than its core ones.

Many authors investigate financial market sectoral connectedness [21,30,31,32,33]. For example, Chatziantoniou et al. [32] find that financials, industry or basic materials are net risk transmitters, while IT, consumer goods, healthcare or telecommunications receive most of the risk. Billio et al. [30] conclude that the banking sector is the main risk driver among financial industries. From our knowledge, no other study has until now studied systemic risk in the European Union, in an entropy-based framework, from the perspective of industries other than financials. We bridge this gap by assessing systemic risk of 24 component industries of Stoxx Europe 600 index. Our work follows into the footsteps of Billio et al. [6] in what concerns the conditional capital shortfall methods used.

## 3. Materials and Methods

As input data, we employ daily returns for all the companies included in the Stoxx 600 index for the January 1st 2007–July 31st 2020 time frame. We opt for this period so as to obtain the longest possible time series for the largest possible number of companies in the index. These companies are divided into 24 industry groups which are presented in Table A1 of Appendix A, following the GICS 24 categorization system.

**Figure A1:**
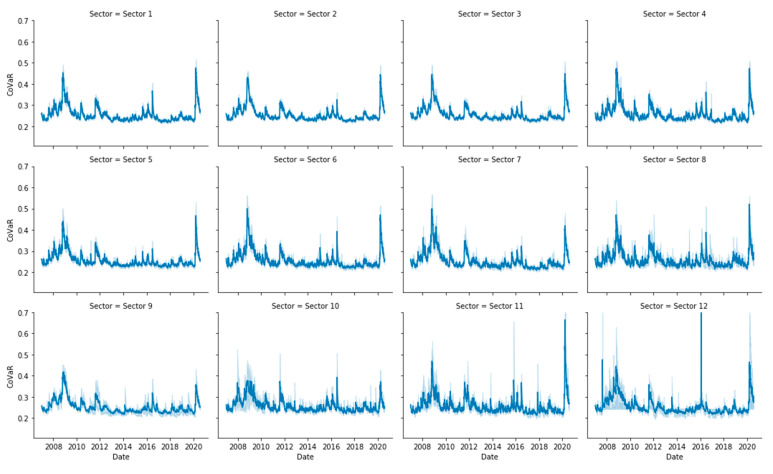

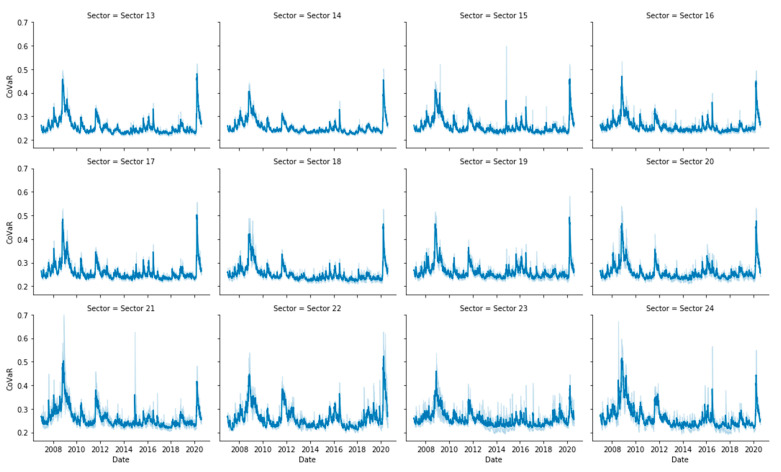
CoVaR measures for all companies across sectors.

**Figure A2:**
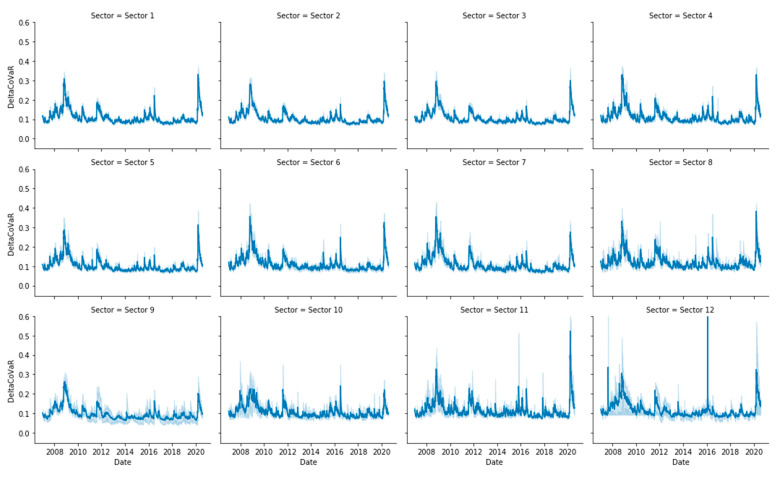

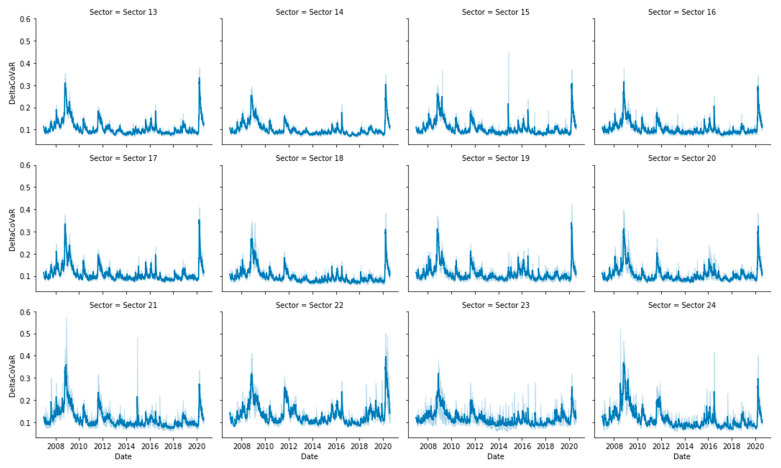
Delta CoVaR measures for all companies across sectors.

**Figure A3:**
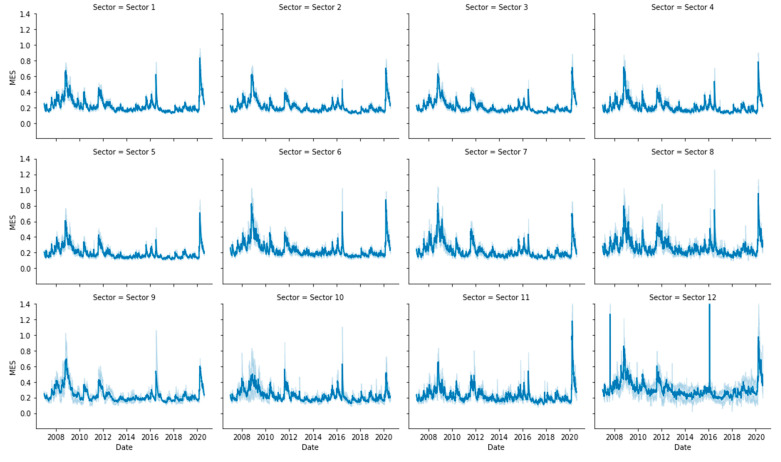

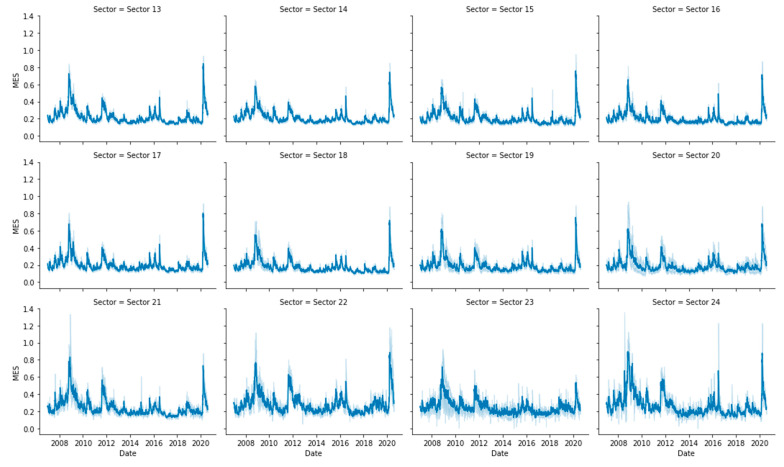
MES measures for all companies across sectors.

We filter the data series for the 600 companies and eliminate those for which we have missing values in more than 10% of the cases. Out of the initial sample, we are left with 500 companies after the filtering procedure. On this filtered batch, we apply the CoVaR, Delta CoVaR, and marginal expected shortfall (MES) measures. Our preliminary results for these measures are shown in Figure A1, Figure A2 and Figure A3 of Appendix B.

**Figure A4:**
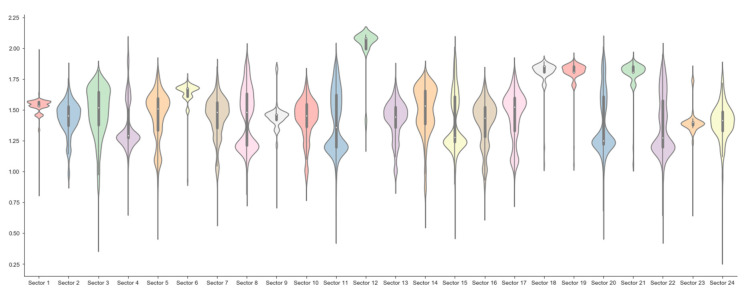
CoVaR-entropies computed for each sector.

**Figure A5:**
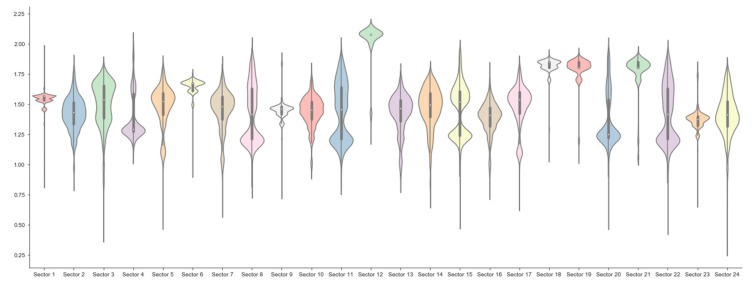
Delta CoVaR-entropies computed for each sector.

**Figure A6:**
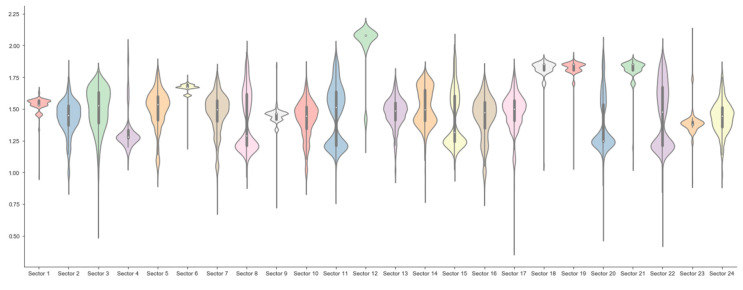
MES-entropies computed for each sector.

From this point, we compute a Bayesian version of the Shannon entropy following the specifications of Archer, Park, and Pillow [34]. We thus obtain entropy values for every moment and every economic sector of the Stoxx 600 index, for each of the three systemic risk measures presented above. Our second batch of preliminary results consisting of entropy values are reported in Figure A4, Figure A5 and Figure A6 of Appendix C. Our approach then turns to two types of investigation. Firstly, we apply the Diebold–Yilmaz procedure (hereafter DY) on the entropies computed for each risk measure. For each day in our sample we compute the entropy across all companies in each sector. For instance, for sector Capital Goods we are using 41 companies (obtained after filtering our data), which means that at each point in time (day) we compute the entropy for the 41 values corresponding to systemic risk measures (one entropy value for CoVaR, one for Delta CoVaR and one for MES) for each company.

For reasons of comparability, the data for each day is scaled by extracting the mean and dividing to the range (maximum minus minimum). For the converted data we compute a histogram and use a standard algorithm to decide the number of bins (An estimate of the bin width of the histogram is first computed using the Scott’s normal reference rule ($h=\frac{3.49\hat{\sigma }}{\sqrt[{3}]{n}}$, where $\hat{\sigma }$ is the sample standard deviation and n is the sample size); given the range of the data, the number of bins in the histogram is computed by dividing the range to this bin width.). According to Archer, Park and Pillow [34] the entropy estimator is computed based on the concept of multiplicity, which consists of two vectors of the same length containing the number of symbols with the same number of occurrences and the corresponding number of occurrences, respectively.

Given the fact that we analyze a cross-sectional distribution of systemic risk measures inside a sector, we consider that the proper prior for the estimation of entropy is one with light tails, which does not impose a large dispersion of data at a particular point in time. The function used as prior is the following:

(1) $$f\left(g\right)=e^{\frac{-10}{1-g}}$$

Details about the computation of the risk measures, the Bayesian entropy values and the spillover index are presented in the subsequent three sub-sections.

### 3.1. Systemic Risk Measures

We first employ the CoVaR and Delta CoVaR measures introduced by Adrian and Brunnermeier (2016). We start from the formulation specific to the value-at-risk of a certain $X^{i}$ institution and a specified quantile *q*.

(2) $$Pr\left(X^{i}\leq VaR_{q}^{i}\right)=q\%$$

where $X^{i}$ denotes the return loss for a certain institution *i*. Under this specification $VaR_{q}^{i}$ is in general a positive number if *q* is above 50.

Adrian and Brunnermeier [35] call by $CoVaR_{q}^{j|C\left(X^{i}\right)}$ the Var of a certain institution *j* (or even the financial system) conditional on a particular event $C\left(X^{i}\right)$ endemic to institution *i*. In other words, $CoVaR_{q}^{j|C\left(X^{i}\right)}$ is given by the *q*%-quantile of the conditional probability distribution. Formally, this can be expressed as:

(3) $$Pr\left(X^{j}|C\left(X^{i}\right)\leq CoVaR_{q}^{j|C\left(X^{i}\right)}\right)=q\%$$

Moreover, Adrian and Brunnermeier [35] isolate *j*’s share of systemic risk that derives from *i* by:

(4) $$\Delta CoVaR_{q}^{j|i}=CoVaR_{q}^{j|X^{i}=Var_{q}^{i}}-CoVaR_{q}^{j|X^{i}=Var_{50}^{i}}$$

In another seminal paper for the field of systemic risk, Acharya et al. [36] remark that it is possible to isolate the systemic risk contribution of a certain institution with the help of an innovative construct called *systemic expected shortfall* (SES). In essence, SES is based on two separate measures, namely the marginal expected shortfall (MES) and leverage (LVG). We focus on the former which by the definition offered by Acharya et al. [36] is the average return during the most deficient 5% days for the market return, where the proxy for the market is the *CRSP Value Weighted Index*.

(5) $$MES_{5\%}^{B}=\frac{1}{number\;of\;days}\sum_{\left\{t:system\;is\;in\;its\;5\%\;tail\right\}}R_{t}^{b}$$

### 3.2. Bayesian Entropy Estimation

We employ a Bayesian approach for the estimation of entropy inspired by the procedure put forward by Archer, Park, and Pillow [34]. We thus first consider Bayesian least squares estimators in the following manner:

(6) $$\hat{H}\left(x\right)=E\left[H|x\right]=\int H\left(\pi \right)p\left(H|\pi \right)p(\pi |x)d\pi$$

where p(π|x) is the posterior given the prior p(π) and the p(X|π) discrete likelihood. In the above setup:

(7) $$p\left(H|\pi \right)=\delta \left(H+\sum_{i}\pi_{i}log\pi_{i}\right)$$

As remarked by Archer, Park, and Pillow [34] the Dirichlet distribution is a tractable alternative for distributions with known finite alphabet size A. It can be formally expressed as:

(8) $$p_{Dir}\left(\pi \right)\propto \prod_{i=1}^{A}\pi_{i}^{a-1}$$

where *a* stands for a concentration parameter.

To correct certain technical undesirable effects associated with narrow posterior intervals, we incorporate the prior specification formulated by Nemenman et al. [37].

(9) $$p\left(\pi \right)=\int p_{Dir}\left(\pi |a\right)p\left(a\right)da$$

where $p_{Dir}\left(\pi |a\right)$ represents the Dir(a) prior on π. The above expression also incorporates a set of weights (p(a)) deriving from:

(10) $$p\left(a\right)\propto \frac{d}{da}E\left[H|a\right]=A\psi_{1}\left(A_{a}+1\right)-\psi_{1}\left(a+1\right)$$

where E[H|a] represents the expected value of H for a *Dir*(*a*) prior, while $\psi_{1}\left(.\right)$ stands for the tri-gamma function.

From this point, the Bayesian Least Squares estimator can be reformulated as:

(11) $$\begin{array}{c} \hat{H}=E\left[H|x\right]=\iint H\left(\pi \right)p(\pi |x,a)p(a|x)d\pi da\\ \hat{H}=\int E\left[H|x,a\right]\frac{p(x|a)p\left(a\right)}{p\left(x\right)}da \end{array}$$

In the above expression, E[H|x,a] represents the posterior mean for the *Dir*(*a*) prior.

To build priors for the infinite discrete distributions we follow in the footsteps of Archer, Park, and Pillow [34] and consider Dirichlet and Pitman-Yor processes. Given the fact that we have a clear perspective on priors, we can infer given observations. We employ a Pitman-Yor adapted Bayesian least squares estimator which can be expressed as:

(12) $${\hat{H}}_{PY}=E\left[H|x\right]=\int E\left[H|x,d,\alpha \right]\frac{p\left(x|d,\alpha \right)p\left(d,\alpha \right)}{p\left(x\right)}d\left(d,\alpha \right)$$

where E[H|x,d,α] represents the expected posterior entropy for the case of a certain (d,α). In this case, the evidence can be formulated as:

(13) $$p\left(x|d,\alpha \right)=\frac{\left(\prod_{l=1}^{K-1}\left(\alpha +ld\right)\right)\left(\prod_{i=1}^{k}\Gamma \left(n_{i}-d\right)\right)\Gamma \left(1+\alpha \right)}{\Gamma {\left(1-d\right)}^{K}\Gamma \left(\alpha +N\right)}$$

### 3.3. Diebold-Yilmaz Spillover Index Computation

The Diebold and Yilmaz [11] model captures spillovers via a general vector autoregression (VAR) setup, in which latent order-dependent results are removed by Cholesky factor orthogonalization.

We assume an N-variable VAR(p) process, stationary in terms of covariance, given by:

(14) $$x_{t}=\overset{p}{\underset{i=1}{\sum }}\Phi_{i}x_{t-1}+\varepsilon_{t}$$

where ε~(0, Σ) represents a vector of i.i.d disturbances. 

Within this framework, the moving average can be formalized as:

(15) $$x_{t}=\overset{\infty }{\underset{i=0}{\sum }}A_{i}\varepsilon_{t-i}$$

In the above equation, the $A_{i}$ coefficient matrices are characterized by:

(16) $$A_{i}=\Phi_{1}A_{i-1}+\Phi_{2}A_{i-2}+\cdots +\Phi_{p}A_{i-p}$$

From this point, Diebold and Yilmaz [11] formulate the expression of the H-step-ahead forecast error variance decomposition in the following manner:

(17) $$\theta_{ij}^{g}\left(H\right)=\frac{\sigma_{ii}^{-1}\sum_{h=0}^{H-1}{\left(e_{i}^{\prime }A_{h}\sum e_{j}\right)}^{2}}{\sum_{h=0}^{H-1}e{(}_{i}^{\prime }A_{h}\sum A_{h}^{\prime }e_{i})}$$

for H=1, 2, …, .

In the above expression, Σ represents the variance matrix associated with the error vector ε. $\sigma_{ii}$ denotes the standard deviation specific to the error term of the *i*th equation, while $e_{i}$ is the selection vector that holds 1 as the *i*th elements and zeros in the other positions.

In addition to this,

(18) $$\overset{N}{\underset{j=1}{\sum }}\theta_{ij}^{g}\left(H\right)\neq 1$$

Each element of the variance decomposition matrix is normalized by the row sum as follows:

(19) $${\tilde{\theta }}_{ij}^{g}\left(H\right)=\frac{\theta_{ij}^{g}\left(H\right)}{\sum_{j=1}^{N}\theta_{ij}^{g}\left(H\right)}$$

As stipulated by Diebold and Yilmaz (2012) specify that by construction:

(20) $$\overset{N}{\underset{j=1}{\sum }}{\tilde{\theta }}_{ij}^{g}\left(H\right)=1$$

and

(21) $$\overset{N}{\underset{i,j=1}{\sum }}{\tilde{\theta }}_{ij}^{g}\left(H\right)=N$$

On the basis of the variance composition, Diebold and Yilmaz [11] put forward first their total volatility spillover index:

(22) $$S^{g}\left(H\right)=\frac{\sum_{\begin{array}{c} i,j=1\\ i\neq j \end{array}}^{N}{\tilde{\theta }}_{ij}^{g}\left(H\right)}{\sum_{i,j=1}^{N}{\tilde{\theta }}_{ij}^{g}\left(H\right)}\cdot 100=\frac{\sum_{\begin{array}{c} i,j=1\\ i\neq j \end{array}}^{N}{\tilde{\theta }}_{ij}^{g}\left(H\right)}{N}\cdot 100$$

The authors also provide a measure of directional volatility spillovers. For example, the directional volatility spillovers obtained by market *i* from market *j* is given by:

(23) $$S_{i\cdot }^{g}\left(H\right)=\frac{\sum_{\begin{array}{c} j=1\\ j\neq i \end{array}}^{N}{\tilde{\theta }}_{ij}^{g}\left(H\right)}{\sum_{j=1}^{N}{\tilde{\theta }}_{ij}^{g}\left(H\right)}\cdot 100$$

In a symmetric way, the directional volatility spillovers induced by market *i* to all other markets *j* is given by:

(24) $$S_{\cdot i}^{g}\left(H\right)=\frac{\sum_{\begin{array}{c} j=1\\ j\neq i \end{array}}^{N}{\tilde{\theta }}_{ji}^{g}\left(H\right)}{\sum_{j=1}^{N}{\tilde{\theta }}_{ji}^{g}\left(H\right)}\cdot 100$$

Given the two equations presented above, the net spillovers, or in other words, the net volatility spillover deriving from market *i* to all other markets *j* is formulated in the following manner:

(25) $$S_{i}^{g}\left(H\right)=S_{\cdot i}^{g}\left(H\right)-S_{i\cdot }^{g}\left(H\right)$$

## 4. Results and Discussion

To approach the main purpose of the article we build on two sets of preliminary results. Appendix B and Appendix C show our computations for the risk measures and the entropies. Given the orientation of our manuscript and space constraints, we do not aim to provide an exhaustive discussion on these preliminary results. In the current section, we provide our main battery of results. First of all, we implement a static DY formulation which is computed on the entire timeframe of our sample. This is conducted for all our systemic risk measures described above, namely CoVaR, delta CoVaR, and MES. Secondly, we opt for the development of a contagion index for the same risk measures. It is constructed via a rolling window approach containing 100 days. Our modeling approach allows for showing graphical representations in the form of heatmaps for each of these 100 days windows which we cannot report due to space constraints, given the length of our sample.

In a subsequent section, we provide details on our robustness exercises. We do this testing first using two classical risk measures by recomputing our entire procedure for the case of beta and value at risk. Secondly, we provide two alternatives for the length of the rolling window specific to the contagion index, recalibrating our baseline models for 200 and 120 days.

### 4.1. Static DY Approach

We now turn our attention to our main block of results, obtained under the static DY approach as explained earlier. We discuss our findings for each of the three systemic risk measures incorporated in our methodology. We follow this procedure in order to provide an early in situ image on the robustness of our results which will also be considered in a separate subsection. Graphically, we incorporate for each of the risk measures three types of representations: a heatmap, a to–from chart and a network chart aiming at showing the relationships that exist among the 24 sectors included in our sample (Due to space constraints we cannot provide an exhaustive discussion about the specifics of each sector. However, our full results are available on demand.).

**Figure 1:**
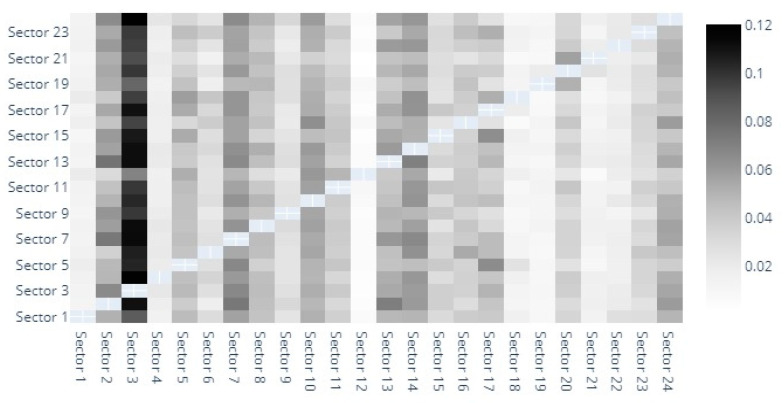
MES heatmap. 1. Automobiles & Components, 2. Banks, 3. Capital Goods, 4. Commercial & Professional Services, 5. Consumer Durables & Apparel, 6. Consumer Services, 7. Diversified Financials, 8. Energy, 9. Food & Staples Retailing, 10. Food, Beverage & Tobacco, 11. Health Care Equipment & Services, 12. Household & Personal Products, 13. Insurance, 14. Materials, 15. Media & Entertainment, 16. Pharmaceuticals, Biotechnology & Life Sciences, 17. Real Estate, 18. Retailing, 19. Semiconductors & Semiconductor Equipment, 20. Software & Services, 21. Technology Hardware & Equipment, 22. Telecommunication Services, 23. Transportation, 24. Utilities.

The heatmaps provide a graphic depiction of the direct spillover indices between sectors. Out of space constraints, we have numbered the sectors and detailed them in the footnotes and Appendix A. In Figure 1 we observe the direct spillover indices based on MES estimates. The darkest shaded sector seems to be that of capital goods, followed by banks, diversified financials, insurance and materials. The capital goods sector definitely stands out as the main entropy sender and receiver. Although this is unusual at first glance, it is noteworthy to remember that our analysis encompasses risk generating industries that have generally been overlooked in previous studies. Transactions with such goods are indeed sensitive to market sentiment, investment dynamics, hence the evolution of the sector being correlated with that of the market. In fact, it is somewhat natural for the capital goods sector to show a high volatility, with quite wide fluctuations. Our finding is actually in line with Collet and Ielpo [38]. However, they use a broader classification of industries, and identify “goods”, along with insurance, as main volatility spillover sources in turbulent times.

The banking sectors also displays strong bidirectional spillovers with the diversified financials, insurance and capital goods sectors. Moreover, the Diversified Financials sector also seems to be closely intertwined with the insurance, and even real estate sectors. If the triangle banking-financials-insurance is widely acknowledged as a risk driver [30,39,40,41,42], far fewer studies have explored risk spillover effects between other industries in such detail as we do. For example, Michail and Savvides [43] find a very strong relation between baking crises and capital goods imports and agree on the pivotal role of capital stock for economic growth.

Lower spillover indices correspond to the following sectors: household and personal products, semiconductors, commercial and professional services, retailing or even automobiles and components.

**Figure 2:**
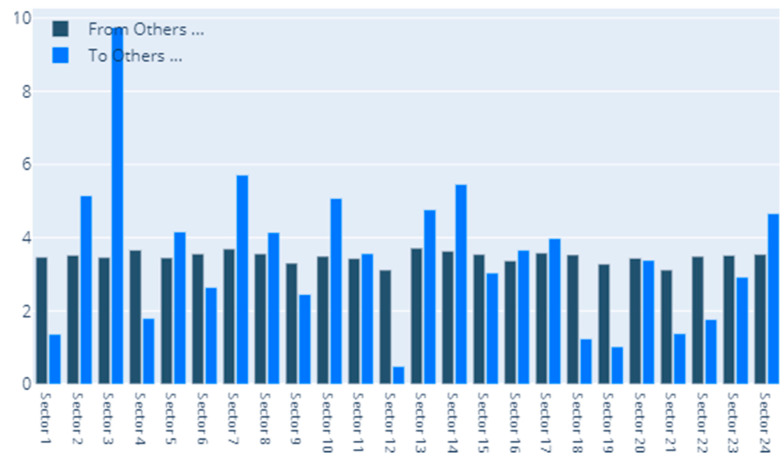
MES: To–From. 1. Automobiles & Components, 2. Banks, 3. Capital Goods, 4. Commercial & Professional Services, 5. Consumer Durables & Apparel, 6. Consumer Services, 7. Diversified Financials, 8. Energy, 9. Food & Staples Retailing, 10. Food, Beverage & Tobacco, 11. Health Care Equipment & Services, 12. Household & Personal Products, 13. Insurance, 14. Materials, 15. Media & Entertainment, 16. Pharmaceuticals, Biotechnology & Life Sciences, 17. Real Estate, 18. Retailing, 19. Semiconductors & Semiconductor Equipment, 20. Software & Services, 21. Technology Hardware & Equipment, 22. Telecommunication Services, 23. Transportation, 24. Utilities.

**Figure 3:**
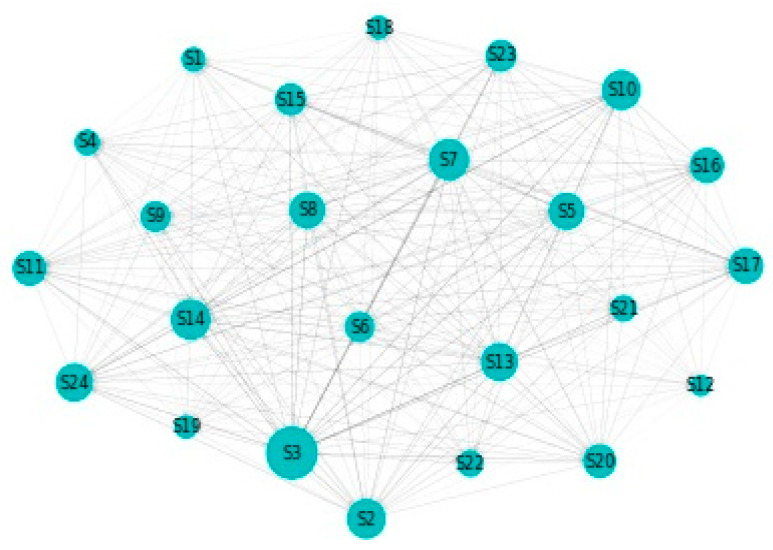
MES: Network. 1. Automobiles & Components, 2. Banks, 3. Capital Goods, 4. Commercial & Professional Services, 5. Consumer Durables & Apparel, 6. Consumer Services, 7. Diversified Financials, 8. Energy, 9. Food & Staples Retailing, 10. Food, Beverage & Tobacco, 11. Health Care Equipment & Services, 12. Household & Personal Products, 13. Insurance, 14. Materials, 15. Media & Entertainment, 16. Pharmaceuticals, Biotechnology & Life Sciences, 17. Real Estate, 18. Retailing, 19. Semiconductors & Semiconductor Equipment, 20. Software & Services, 21. Technology Hardware & Equipment, 22. Telecommunication Services, 23. Transportation, 24. Utilities.

Figure 2 details our to–from computation of spillovers. In terms of spillovers to a specific sector, our highest result is for sector 3, namely Capital Goods. An interesting observation is that the spillovers to the Capital Goods sector have a magnitude that greatly surpasses other similar situations. Other sectors that manifest high spillovers in their direction are: Banks, Diversified Financials, Food, Beverage and Tobacco, Insurance, Materials and Utilities. In other words, these are the sectors that are the most vulnerable to spillovers. This is partially in line with the findings of Chatziantoniou et al. [32], who provide evidence that financials, basic materials and consumer spending are the major risk generating industries. On the opposite spectrum, the lowest influences are noticed for economic sectors such as Retailing, Automobiles & Components or Technology Hardware & Equipment. The other side of this specific analysis reveals the sectors that induce the most spillovers into the system. Our results show that Insurance, Diversified Financials, Commercial & Professional Services and Real Estate generate the most relevant effects. This is in line with previous literature and consistent with normal financial intuition. For example, Wang et al. [39] also find insurance, real estate and diversified financials to be main spillover drivers in financial markets. Chan et al. [44], Zhou and Gao [45] or Bouri, Gupta and Wang [46] find that the real estate sector is highly volatile and contagion prone. Moreover, He, Liu and Chen [47] argue that the spillover dynamics of real estate and financials represent a very strong warning sign of systemic risk. All the relations existing in our results are presented graphically in Figure 3. As a rule of thumb, for the network charts included in this section, the size of a certain section in the graphical representation is given by its overall impact detected in the results.

**Figure 4:**
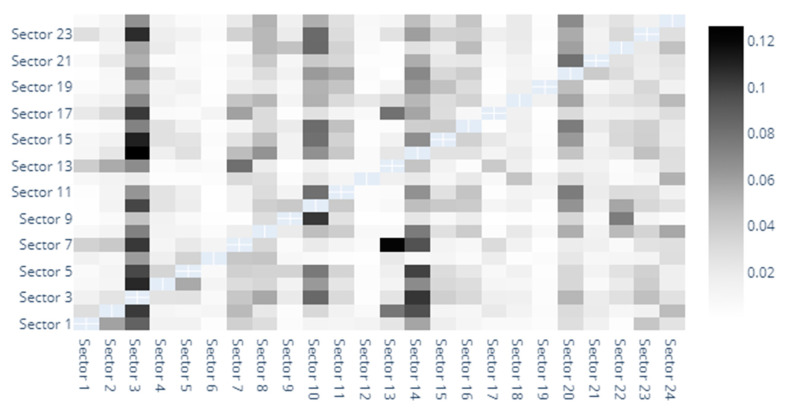
CoVaR heatmap. 1. Automobiles & Components, 2. Banks, 3. Capital Goods, 4. Commercial & Professional Services, 5. Consumer Durables & Apparel, 6. Consumer Services, 7. Diversified Financials, 8. Energy, 9. Food & Staples Retailing, 10. Food, Beverage & Tobacco, 11. Health Care Equipment & Services, 12. Household & Personal Products, 13. Insurance, 14. Materials, 15. Media & Entertainment, 16. Pharmaceuticals, Biotechnology & Life Sciences, 17. Real Estate, 18. Retailing, 19. Semiconductors & Semiconductor Equipment, 20. Software & Services, 21. Technology Hardware & Equipment, 22. Telecommunication Services, 23. Transportation, 24. Utilities.

**Figure 5:**
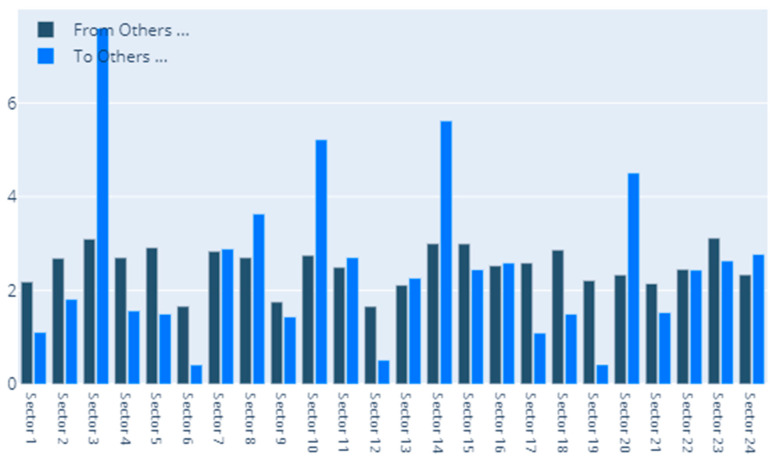
CoVaR: To–From. 1. Automobiles & Components, 2. Banks, 3. Capital Goods, 4. Commercial & Professional Services, 5. Consumer Durables & Apparel, 6. Consumer Services, 7. Diversified Financials, 8. Energy, 9. Food & Staples Retailing, 10. Food, Beverage & Tobacco, 11. Health Care Equipment & Services, 12. Household & Personal Products, 13. Insurance, 14. Materials, 15. Media & Entertainment, 16. Pharmaceuticals, Biotechnology & Life Sciences, 17. Real Estate, 18. Retailing, 19. Semiconductors & Semiconductor Equipment, 20. Software & Services, 21. Technology Hardware & Equipment, 22. Telecommunication Services, 23. Transportation, 24. Utilities.

**Figure 6:**
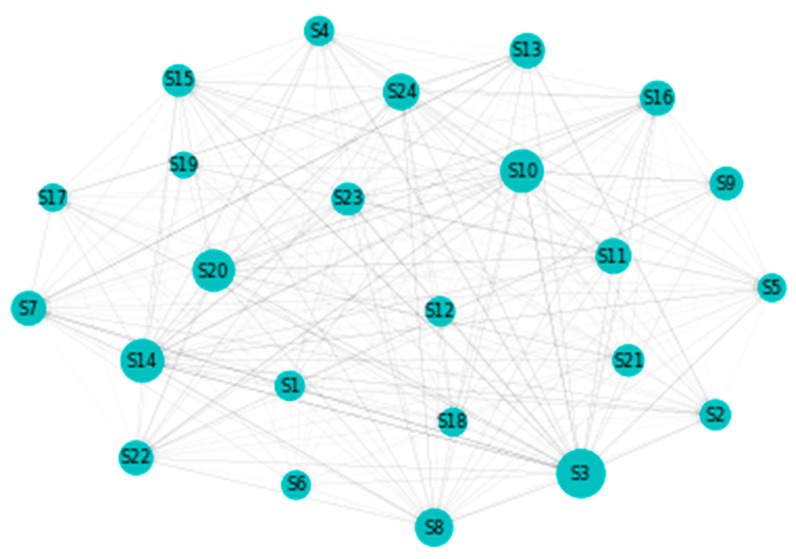
CoVaR: Network. 1. Automobiles & Components, 2. Banks, 3. Capital Goods, 4. Commercial & Professional Services, 5. Consumer Durables & Apparel, 6. Consumer Services, 7. Diversified Financials, 8. Energy, 9. Food & Staples Retailing, 10. Food, Beverage & Tobacco, 11. Health Care Equipment & Services, 12. Household & Personal Products, 13. Insurance, 14. Materials, 15. Media & Entertainment, 16. Pharmaceuticals, Biotechnology & Life Sciences, 17. Real Estate, 18. Retailing, 19. Semiconductors & Semiconductor Equipment, 20. Software & Services, 21. Technology Hardware & Equipment, 22. Telecommunication Services, 23. Transportation, 24. Utilities.

Figure 4, Figure 5 and Figure 6 describe the results obtained for the CoVaR measure in a manner similar to the one provided above. The results on which Figure 4 is built manifest several relevant take-aways. First of all, we notice from the start that sectors 3, 14, and 20 generate spillovers in relation to a relevant number of other sectors. The most consistent effect is visible for sector 3, namely Capital Goods. Our findings suggest that this sector generates spillovers to all other sectors besides Food and Staples Retailing and Household and Personal Products. This is consistent with the results for our previous specification and with the conclusions provided by Collet and Ielpo [38]. In addition to this, we detect consistent spillovers between Diversified Financials and Banks, Insurance, Materials and Real Estate.

We also obtain results that can be considered in line with normal economic intuition. For example, there are consistent spillovers between the Energy sector and other economic sectors such as Utilities, Transportation, Retailing or Materials.

We now turn our attention to the to–from analysis which is visible for CoVaR in Figure 5. In a similar manner to the MES specification, in this area we consider the sectors that are more prone to attracting and generating spillovers in the system. We remark that the sectors that feel the most profound influence in terms of spillovers are Capital Goods, Materials, Food, Beverage and Tobacco, Software and Services and Energy. In the same time, the sectors that manifest the lowest impact are Consumer Services, Semiconductors and Semiconductor Equipment and Household and Personal Products. Along the same line, the sectors that generate the most amount of spillover are Transportation, Capital Goods, and Materials. Central positions are occupied also by Banks and Diversified Financials. All the above interactions are also visible in Figure 6.

**Figure 7:**
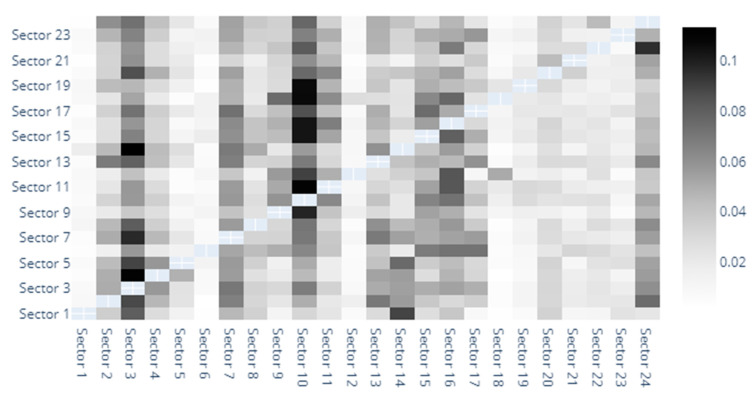
Delta CoVaR heatmap. 1. Automobiles & Components, 2. Banks, 3. Capital Goods, 4. Commercial & Professional Services, 5. Consumer Durables & Apparel, 6. Consumer Services, 7. Diversified Financials, 8. Energy, 9. Food & Staples Retailing, 10. Food, Beverage & Tobacco, 11. Health Care Equipment & Services, 12. Household & Personal Products, 13. Insurance, 14. Materials, 15. Media & Entertainment, 16. Pharmaceuticals, Biotechnology & Life Sciences, 17. Real Estate, 18. Retailing, 19. Semiconductors & Semiconductor Equipment, 20. Software & Services, 21. Technology Hardware & Equipment, 22. Telecommunication Services, 23. Transportation, 24. Utilities.

**Figure 8:**
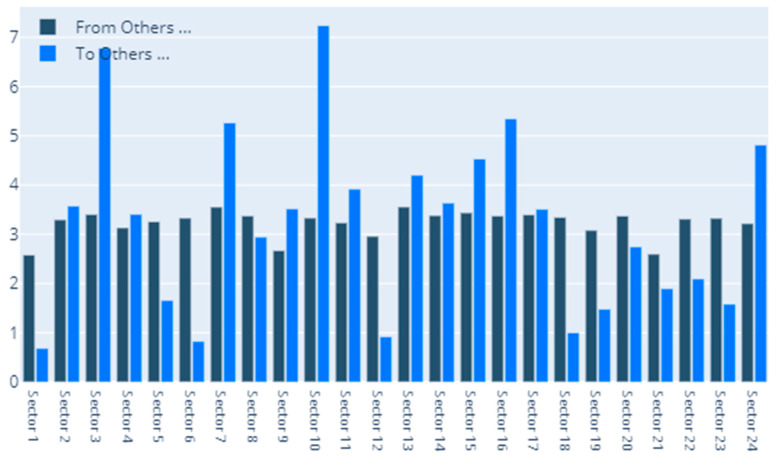
Delta CoVaR: To–From. 1. Automobiles & Components, 2. Banks, 3. Capital Goods, 4. Commercial & Professional Services, 5. Consumer Durables & Apparel, 6. Consumer Services, 7. Diversified Financials, 8. Energy, 9. Food & Staples Retailing, 10. Food, Beverage & Tobacco, 11. Health Care Equipment & Services, 12. Household & Personal Products, 13. Insurance, 14. Materials, 15. Media & Entertainment, 16. Pharmaceuticals, Biotechnology & Life Sciences, 17. Real Estate, 18. Retailing, 19. Semiconductors & Semiconductor Equipment, 20. Software & Services, 21. Technology Hardware & Equipment, 22. Telecommunication Services, 23. Transportation, 24. Utilities.

**Figure 9:**
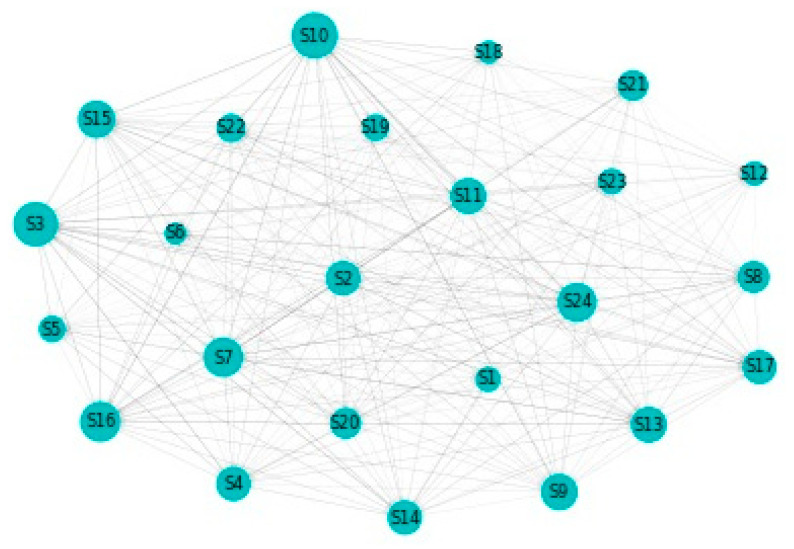
Delta CoVaR: Network. 1. Automobiles & Components, 2. Banks, 3. Capital Goods, 4. Commercial & Professional Services, 5. Consumer Durables & Apparel, 6. Consumer Services, 7. Diversified Financials, 8. Energy, 9. Food & Staples Retailing, 10. Food, Beverage & Tobacco, 11. Health Care Equipment & Services, 12. Household & Personal Products, 13. Insurance, 14. Materials, 15. Media & Entertainment, 16. Pharmaceuticals, Biotechnology & Life Sciences, 17. Real Estate, 18. Retailing, 19. Semiconductors & Semiconductor Equipment, 20. Software & Services, 21. Technology Hardware & Equipment, 22. Telecommunication Services, 23. Transportation, 24. Utilities.

The last model in our baseline specification is the one built around the Delta CoVaR systemic risk measure under the previsions described in our methodology. Figure 7, Figure 8 and Figure 9 aim to delineate the results obtained at this point. We start from considering the spillovers as portrayed in the heat map offered in Figure 7. Our findings reveal from the start that several sectors are extremely efficient in generating spillovers to their counterparts. Good examples in this direction are sector 3 (Capital Goods), sector 7 (Diversified Financials), sector 10 (Food, Beverage, and Tobacco), sector 13 (Insurance), sector 14 (Materials), sector 17 (Real Estate) and sector 24 (Utilities). This is in line with the results obtained in the above sections and especially close to the findings obtained under the MES specification, fact that can be regarded as a measure of results’ robustness, despite the fact that this aspect will be covered later on in the manuscript.

Moreover, we notice for this modeling specification as well as relevant spillover effects between Banks and sectors such as Capital Goods, Commercial and Professional Services, Diversified Financials, and Insurance. In addition to this, we remark spillovers between Diversified Financials and 19 out of the 24 sectors in our sample.

Figure 8 sheds light on the to-from analysis for the case of the delta CoVaR specification. We notice that the sectors that are the most vulnerable in terms of receiving spillover effects are Food, Beverage & Tobacco (sector 10), Capital Goods (sector 3), Pharmaceuticals, Biotechnology &Life Sciences (sector 16), and Diversified Financials. The Insurance sector also places quite high in this ranking. Staying in relation to the Insurance sector, we detect the fact that it represents the largest contributor of spillovers to the other sectors. The second position is held by Diversified Financials, while Capital Goods is forth in this ranking after Media & Entertainment. Finally, Figure 9 shows the interconnections between all of the sectors in our sample. As noted above, the size of a certain sector in the graphical representation portrays its overall influence.

### 4.2. Rolling Window DY Approach

In this section, we discuss the results for our second type of analysis, namely the rolling window DY approach. It allows us to determine the dynamics of a contagion index across all economic sectors for each of the systemic risk measures employed. Similar approaches or modeling variants have been used in financial or economic applications during the recent period.

For example, in a very recent contribution, Akhtaruzzaman et al. [48] try to make a parallel between the US and China as a regional contagion transmitter. The main contribution of the article is the fact that both US and Chinese firms transmitted more spillovers than they sustained during the global financial crisis. Other relevant and à la mode investigations can be traced to Gong et al. [49] or Corbet et al. [50].

**Figure 10:**
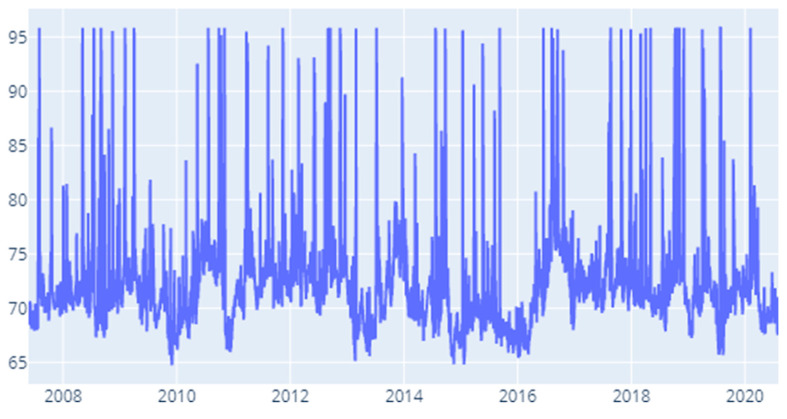
MES: Time-varying spillover index.

**Figure 11:**
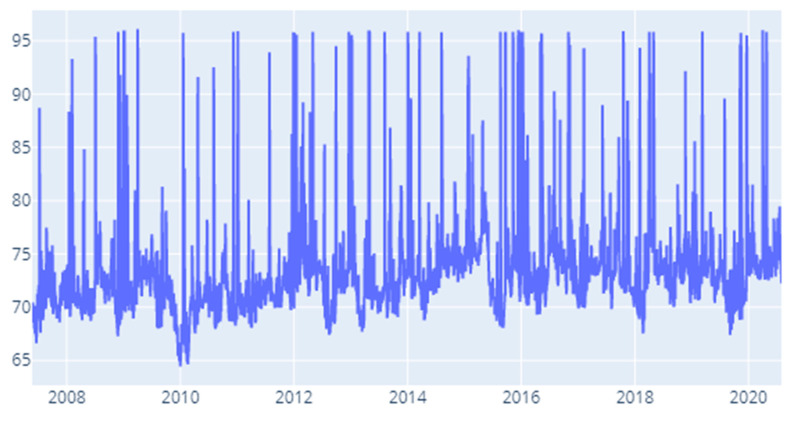
CoVaR: Time-varying spillover index.

**Figure 12:**
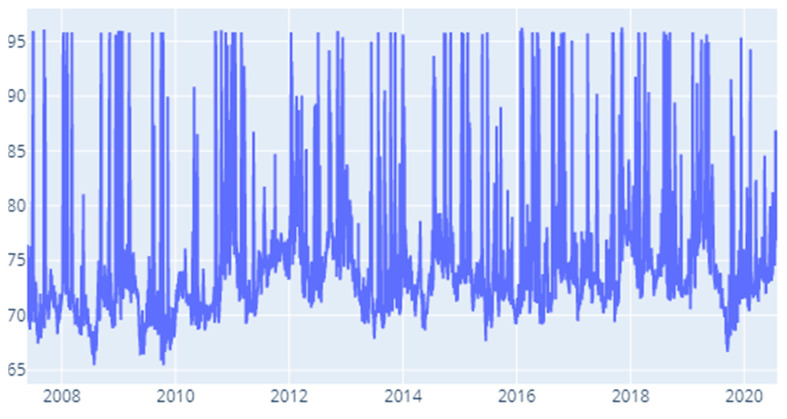
Delta CoVaR: Time-varying spillover index.

Figure 10, Figure 11 and Figure 12 relay the results for the three systemic risk measures employed in our methodology.

Under the MES specification (Figure 10) we notice a concentration of high values for the contagion index (filtered by the rule of the highest 20% of values) in 2012 Q1, Q1 and Q4. In addition to this, we remark high values for the ending quarters of 2008 (Q3), 2010 (Q3), 2012 (Q3, Q4), 2013 (Q4), 2016 (Q3), 2017 (Q3, Q4), and 2018 (Q4). Though it does not fall under the scope of the present investigation, these results mark in interesting pattern that could be expanded in future research.

The results obtained for the CoVaR measure (Figure 11) paint an interesting picture. We notice that the largest values for the contagion index are obtained for the 2012–2020 interval. More exactly, the largest 20% of values are distributed to the following time spans: January–May 2012, August–November 2014, January–May 2015, November 2015–January 2016, July–November 2016, the second quarter of 2018 and quarters 2 and 3 of 2020. Conversely, we notice the lowest values for 2010 Q1 to Q3 and the middle quarters of 2011 and 2012. A challenging research question that derives from these results resides in investigating the drivers of this inter-sector contagion around the above moments.

Our final specification is built around the delta CoVaR measure. We notice high contagion in the first quarter of 2008 and then similar results to the precedent risk measures. Notable moments are that hint to contagion pilling up are: 2010 (Q4), 2012, (Q1 and Q4), 2014 (Q3), 2015 (Q1), 2017 (Q4), 2018 (Q3), 2019 (Q2) and 2020 (Q3).

### 4.3. Robustness Testing

After discussing the findings for our base specifications, we turn our attention to robustness testing. This is done using two very well-known risk measures namely beta and value at risk.

**Figure A7:**
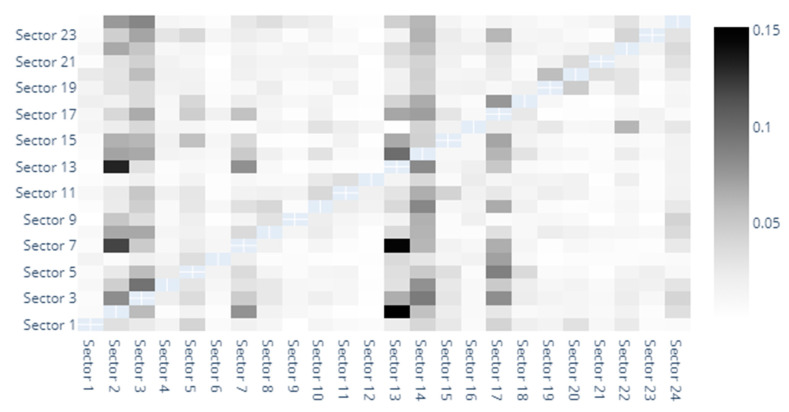
Beta heatmap.

**Figure A8:**
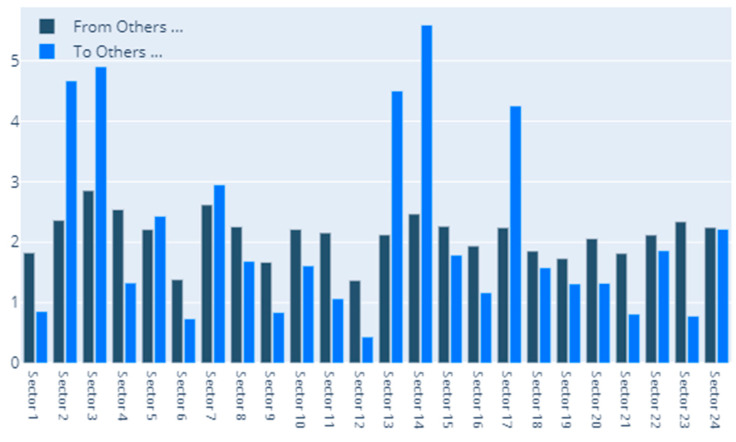
Beta: To–From.

**Figure A9:**
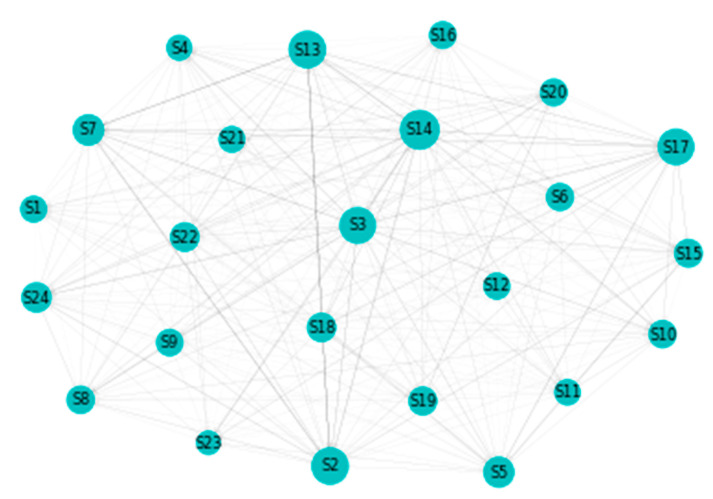
Beta: Network.

**Figure A10:**
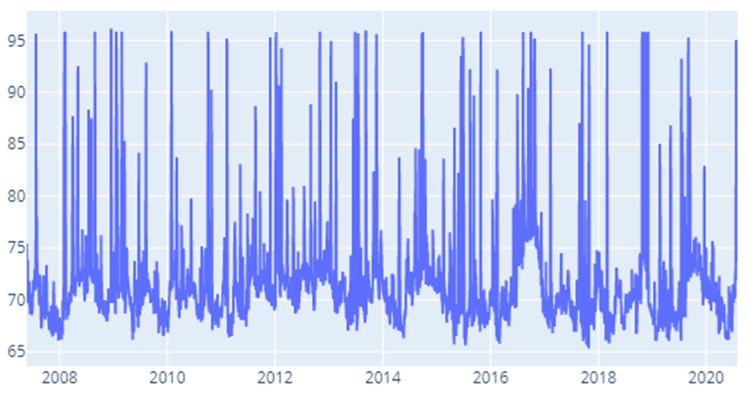
Beta: Time-varying spillover index.

In this section, we do not aim to provide visual representations of the results in the main body but focus more on recalibrating all the previous modeling actions for the case of other measures of risk. However, Appendix D and Appendix E hold the graphical representations of our calculations. We report our findings in the same order we employed for our baseline models. Our first robustness is centered around beta (Figure A7, Figure A8, Figure A9 and Figure A10 from Appendix D). We consider first our static approach and look at the DY results on which we previously constructed heat maps. Our initial observation is that sectors 2 (Banks), 3 (Capital Goods), 5 (Consumer Durables and Apparel), 7 (Diversified Financials), 13 (Insurance), 14 (Materials), and 17 (Real Estate) are extremely efficient in generating spillovers to their counterparts. This is in line with what we obtained for the three systemic risk measures detailed in the previous section. We notice of pattern that determines Banks, Capital Goods, Diversified Financials, and Real Estate to be relevant sectors both in the baseline specifications and in the beta-based framework.

We note the fact that Banks, Capital Goods, Insurance, and Real Estate hold the most relevant impact in the current setup which is close to the results obtained for the MES-based model.

We now consider the to–from spillover analysis for the beta specification. The top five recipient sectors are Materials, Capital Goods, Banks, Insurance, and Real Estate. Antithetically, the top five sectors that generate spillovers are: Capital Goods, Diversified Financials, Commercial and Professional Services, and Banks. We catch sight of the fact that the results fall very close to our baseline models.

The last stop in the beta-based analysis is the rolling window DY procedure. We notice from the beginning a concentration of elevated values for the contagion index in the middle quarters of 2008 and the first quarter of 2009. After this point, we remark that the highest values of the contagion index are located in the final quarters of 2011 (Q3), 2012 (Q3 and Q4), 2013 (Q4), 2014 (Q3, Q4), 2016 (Q3, Q4), 2018 (Q4), 2019 (Q3 and Q4) and finally 2020 (Q3). This is very close to the results obtained for the MES baseline specification.

**Figure A11:**
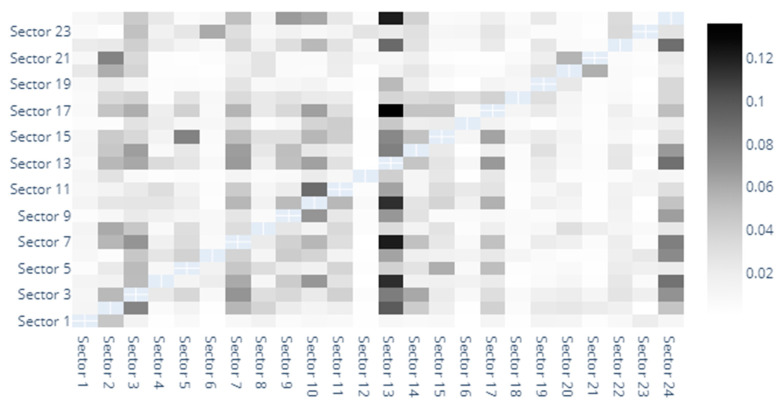
VaR heatmap.

**Figure A12:**
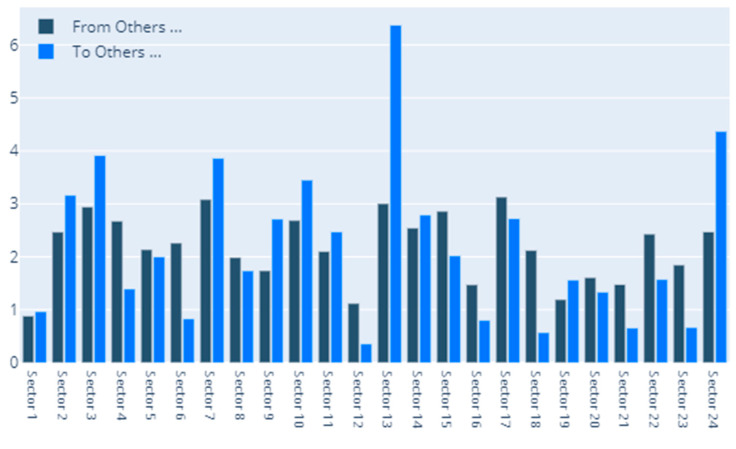
VaR: To–From.

**Figure A13:**
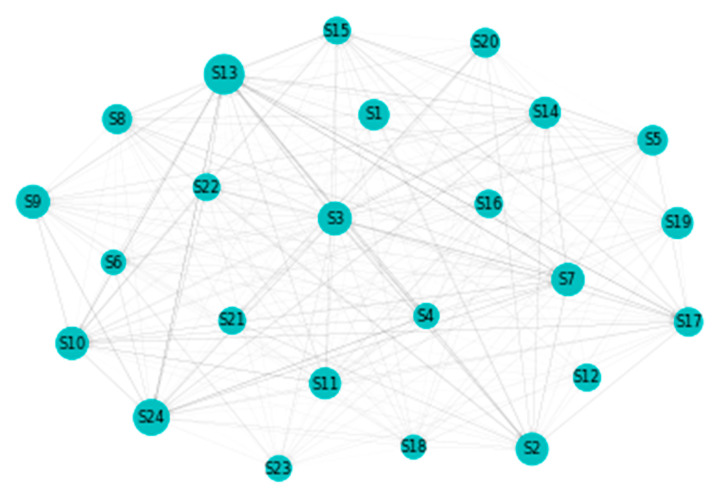
VaR: Network.

**Figure A14:**
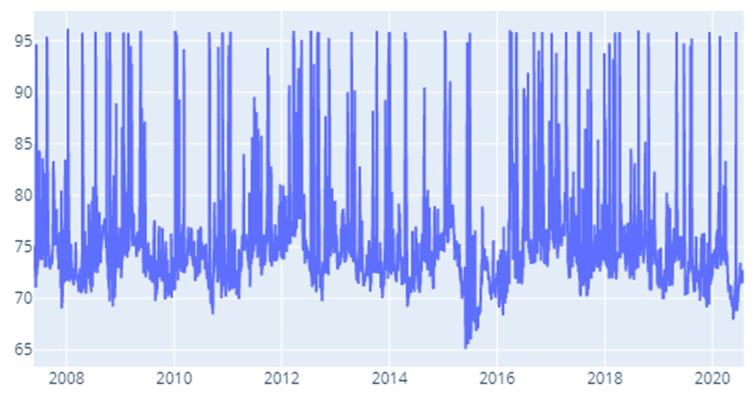
VaR: Time-varying spillover index.

Our second robustness exercise is built around a value at risk model. The results for this specification are reported in Figure A10, Figure A11, Figure A12, Figure A13 and Figure A14, belonging to Appendix E. In the static DY orientation, the results point out consistent spillovers deriving from sectors such as Banks, Capital Goods, Diversified Financials, Insurance, Real Estate, and Utilities. In this modeling setup, though high, the contribution of Banks and Real Estate is far more subtle than in the previous case. This is to a certain extent compensated by a more solid contribution of the Insurance sector.

This is visible to a certain extent also in the to-from investigation. We spot the fact that Insurance represents the sector that is most vulnerable to spillovers. It is followed by Utilities, Capital Goods, and Diversified Financials.

Despite the above-mentioned results, we notice that Real Estate represents the sector that generates the most spillovers. It is closely followed by Diversified Financials, Insurance, and Capital Goods.

We also compute the contagion index under the rolling window DY approach for the value at risk specification. Similar to the previous estimation, we obtain high values for the end of 2008 and the beginning of 2009. We remark concentrations of consistent values in 2012 (Q1–Q3), in the last quarters of 2013 and 2014, and in 2016 (Q2–Q4). Despite a heterogenous character, the results obtained under the specification catered on the value at risk measure manifest a high degree of similarity to the baseline specifications.

**Figure A15:**
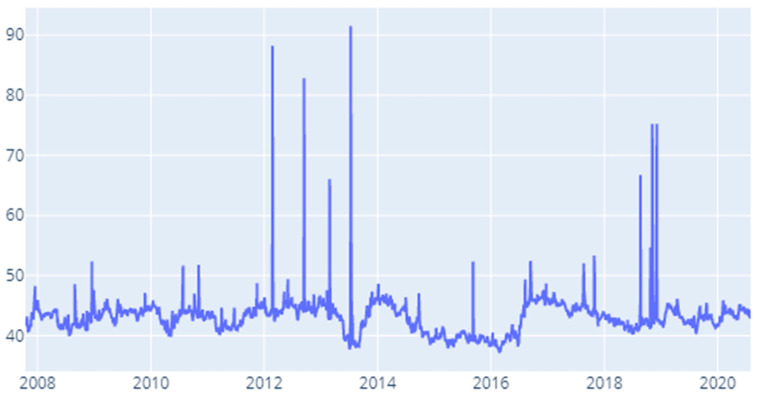
MES: Time-varying spillover index—200 days.

**Figure A16:**
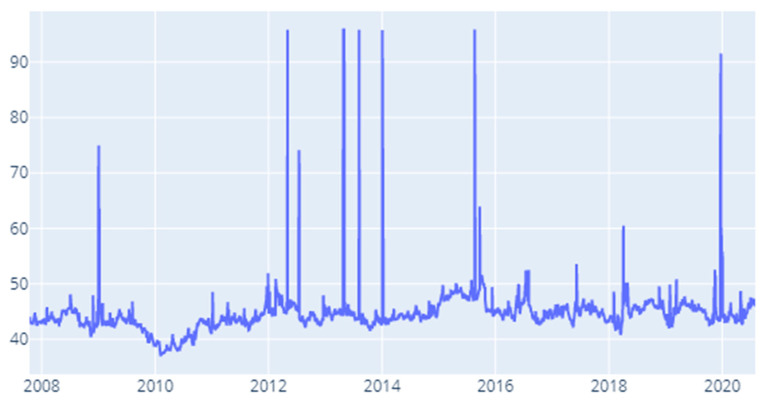
CoVaR: Time-varying spillover index—200 days.

**Figure A17:**
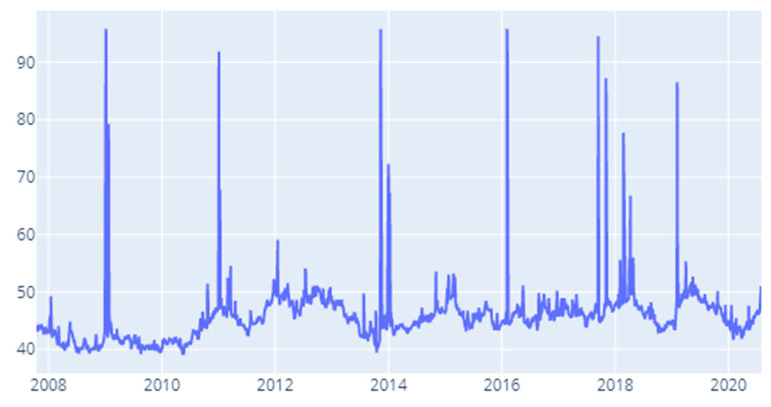
Delta CoVaR: Time-varying spillover index—200 days.

**Figure A18:**
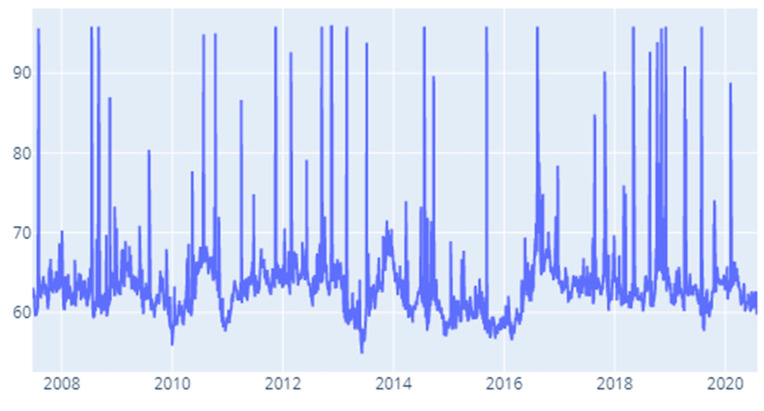
MES: Time-varying spillover index—120 days.

**Figure A19:**
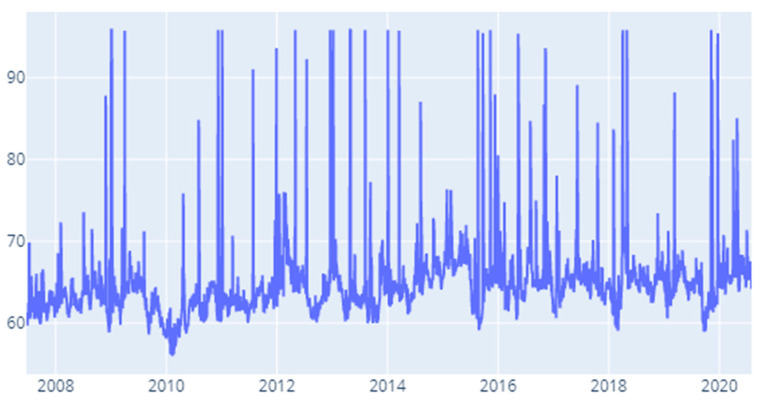
CoVaR: Time-varying spillover index—120 days.

**Figure A20:**
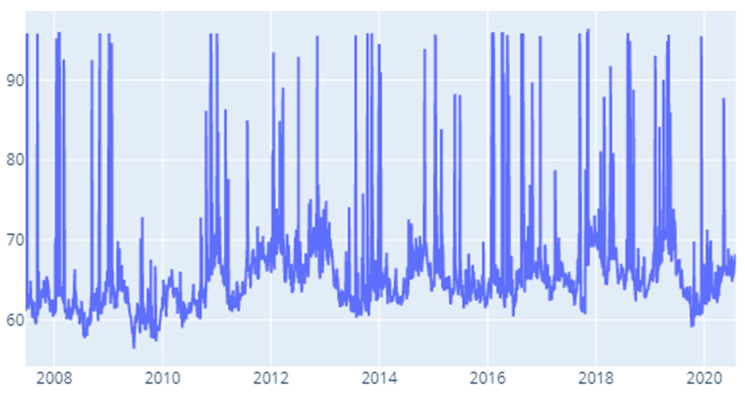
Delta CoVaR: Time-varying spillover index—120 days

We give special attention to our rolling window DY approach in terms of testing for robustness. We do this by re-computing our contagion index for different window lengths than the main specification which relied on a 100-day window. We first chose a 200-day window following in the footsteps of the original contribution put out by Diebold and Yilmaz (2012). Secondly, we re-estimate the contagion index for a rolling window of 120 which corresponds to 6 months of the trading year and we run the recalibration for our three systemic risk measures in our baseline specifications. Our results are summarized in Figure A15, Figure A16, Figure A17, Figure A18, Figure A19 and Figure A20 of Appendix F. Our main observation is that for the 200-day rolling window estimation the results fall close to those obtained for the baseline. For the MES specification, we remark high values of the contagion index in the last months of both 2008 and 2009. However, larger values are noticed during the sovereign debt crisis of the Eurozone, throughout 2012, 2013 (Q1 and Q4), in 2014 (Q1 and Q2), during the short Brexit episode of 2016. Slight increases also occur in 2017 (Q1, Q2, Q3), and the last quarter of 2018.

The 200-day rolling window specification for the CoVaR measure yields comparable results to the baseline model. We detect inflated values for the contagion index in the first two quarters of 2012, the second quarter of 2013, and for all quarters of 2015. Furthermore, the results point to elevated values of contagion for the last three quarters of 2018, 2019 (Q2 and Q4), and 2020 (Q3).

The last systemic risk method included in our analysis is the Delta CoVaR. For this specification, we notice that the top values for the contagion index are firstly detected in 2011 (Q1 and Q4) and throughout 2012. These are followed by 2015 and 2016 (Q1 in both cases), 2017 (Q4), 2018 (Q1 and Q2), and the first three quarters of 2019.

We now turn our attention to the last robustness exercise of the section, which as stated above consists of recomputing the DY rolling contagion index, this time for a window of 120 days. As usual, our first specification is the MES model. In line with both the baseline model and the previous robustness test, we distinguish the fact that this systemic risk measure captures enhanced contagion episodes in the crisis years, more specifically in 2008 Q3 and 2009 Q1. Other periods in which the contagion index manifests high values are 2009 Q1, 2010 Q3, 2011 (Q3 and Q4), 2012 (all quarters), 2013 Q4, 2016 and 2017 (Q3 and Q4), 2018 Q4 and 2020 Q1. Second on our list of systemic risk measures is the CoVaR measure. We notice high levels of contagion in 2009 Q2, 2012 (Q1, Q2), 2013 (Q2), 2014 (Q3, Q4), 2015 (Q1, Q2, Q4), 2016 (Q3, Q4), 2017 and 2018 (Q2) and in the first three quarters of 2020, during the peak of the pandemic crisis. Lastly, when computing the contagion index with the rolling window set as 120 days, we notice accumulations of contagion in 2010 and 2011 Q4, 2012 (Q1, Q4), 2014 Q4, 2015 Q1, 2016 (Q1, Q2, and Q3), 2017 Q4, 2018 (Q1, Q2, and Q3) and 2019 (Q1, Q2). An early conclusion is the fact that our results are generally robust to changes in the rolling window employed for the DY contagion index estimation. Despite the fact that a wider horizon has a smoothing effect on the results, they manifest a high degree of similarity to the baseline specification. We notice that the overall spillover index is generally higher in turbulent times, which is in line with the findings of studies such as: Andrada-Félix et al. [21], Chau and Deesomsak [23], Chatziantoniou et al. [32], Liu and Hamori [33], Barunik et al. [51], Demirer et al. [52], and Marfatia et al. [53].

## 5. Conclusions

The recent financial crisis has clearly shown the extorsions in financial and macroeconomic stability that spun from systemic events. This resulted in a cogent and well-grounded vein of interest deriving from both academia and policy-making circles. Given this dedicated interest in systemic risk measurement, this paper fuses two types of measures in order to scrutinize its transmission across the European capital market. We start from three key systemic risk measures and integrate an entropy construction for each of them in an approach that is related to a certain extent to the work of Billio et al. (2016).

Our contribution is stamped by the fact that we decide to assimilate in our approach all the sectors that map out the Stoxx 600 index without considering only financial institutions. This allows us to perceive the sources of shocks for the entire system. After dealing with intermediary steps endemic to systemic risk measure computation or entropy estimation we turn to two separate approaches. We focus on both a static DY procedure and a rolling window DY outlook. This is done for three baseline specifications and later for two robustness tests. In this setup we get to a series of clear takeaways that derive from the investigation. First of all, we notice that several sectors have a high propensity of generating spillovers. In general, the largest influences are delivered by Capital Goods, Banks, Diversified Financials, Insurance and Real Estate. Secondly, we deliver clear evidence on the sectors that are the most vulnerable to spillovers and on those that represent the main contributors of spillovers. Thirdly we compute a rolling window contagion index and timestamp its values throughout our sample.

Our results are consistent in all baseline specifications and in the robustness testing. In what concerns the overall spillover indices, the results are heterogenous to an extent, but exhibit interesting recurrence patterns. A high similarity can be observed between the MES and delta CoVaR-based spillover measures. For example, both the overall spillover indices of entropy calculated for MES and delta CoVaR identify insurance and diversified financials as the main risk generating industries.

Our findings once again confirm the widely acknowledged systemic relevance of banks, financials, insurance or real estate. Moreover, and perhaps most importantly, we shed light on sectors neglected before as risk spillover drivers that lower the advantage of portfolio diversification. These particular other industries incur higher than expected risks that cannot be neglected by policy makers, market participants and academia. Conversely, the sectors ranking last in the risk transmitting hierarchy may provide good investment alternatives in order to benefit better of diversification opportunities. In other words, our results have policy implications in what concerns risk concentration and risk exposure. From this policy point of view, the insights given by our research may be useful for the setup of macroprudential policies, particularly when establishing risk concentration exposure limits for financial institutions.

## Appendix A. Economic Sectors

**Table A1:** Sectors.

Sectors	
1.	Automobiles & Components
2.	Banks
3.	Capital Goods
4.	Commercial & Professional Services
5.	Consumer Durables & Apparel
6.	Consumer Services
7.	Diversified Financials
8.	Energy
9.	Food & Staples Retailing
10.	Food, Beverage & Tobacco
11.	Health Care Equipment & Services
12.	Household & Personal Products
13.	Insurance
14.	Materials
15.	Media & Entertainment
16.	Pharmaceuticals, Biotechnology &Life Sciences
17.	Real Estate
18.	Retailing
19.	Semiconductors & Semiconductor Equipment
20.	Software & Services
21.	Technology Hardware & Equipment
22.	Telecommunication Services
23.	Transportation
24.	Utilities
